# Multiple mesodermal lineage differentiation of *Apodemus sylvaticus *embryonic stem cells in vitro

**DOI:** 10.1186/1471-2121-11-42

**Published:** 2010-06-19

**Authors:** Tao Wang, Frank Fuxiang Mao, Wenyu Lai, Weiqiang Li, Weihua Yu, Zifei Wang, Lirong Zhang, Jinli Zhang, Jin Niu, Xiuming Zhang, Bruce T Lahn, Andy Peng Xiang

**Affiliations:** 1 Center for Stem Cell Biology and Tissue Engineering, Sun Yat-Sen University, 74# Zhongshan Road 2, Guangzhou, 510080, China; 2 The Key Laboratory for Stem Cells and Tissue Engineering, Ministry of Education; Key Laboratory of Stem Cells and Tissue Engineering of Guangdong Higher Education Institutes, Sun Yat-sen University, Guangzhou, China; 3 Department of Pediatric, The Second Affiliated Hospital of Sun Yat-sen University, Yanjiang Xi Lu, Guangzhou 510120, China; 4 Department of Biochemistry, Zhongshan Medical School, Sun Yat-sen University, Guangzhou, China; 5 Department of Pathophysiology, Guangdong College of Pharmacy, Guangzhou, China; 6 Bellaire High School, Houston, TX, USA

## Abstract

**Background:**

Embryonic stem (ES) cells have attracted significant attention from researchers around the world because of their ability to undergo indefinite self-renewal and produce derivatives from the three cell lineages, which has enormous value in research and clinical applications. Until now, many ES cell lines of different mammals have been established and studied. In addition, recently, AS-ES1 cells derived from *Apodemus sylvaticus *were established and identified by our laboratory as a new mammalian ES cell line. Hence further research, in the application of AS-ES1 cells, is warranted.

**Results:**

Herein we report the generation of multiple mesodermal AS-ES1 lineages via embryoid body (EB) formation by the hanging drop method and the addition of particular reagents and factors for induction at the stage of EB attachment. The AS-ES1 cells generated separately in vitro included: adipocytes, osteoblasts, chondrocytes and cardiomyocytes. Histochemical staining, immunofluorescent staining and RT-PCR were carried out to confirm the formation of multiple mesodermal lineage cells.

**Conclusions:**

The appropriate reagents and culture milieu used in mesodermal differentiation of mouse ES cells also guide the differentiation of in vitro AS-ES1 cells into distinct mesoderm-derived cells. This study provides a better understanding of the characteristics of AS-ES1 cells, a new species ES cell line and promotes the use of Apodemus ES cells as a complement to mouse ES cells in future studies.

## Background

Embryonic stem (ES) cells are pluripotent cells derived from the inner cell mass of blastocyst-stage embryos [1]. The abilities of ES cells to undergo indefinite self-renewal in vitro and to produce derivative lineages of all three embryonic germ layers in vitro and in vivo make them highly prized in both clinical and research settings [2]. ES, or ES-like, cells have thus far been derived from a number of mammalian species, including the mouse [3], rat [4], bovine [5], sheep [6], pig [7], rhesus macaque [8], crab-eating macaque [9], marmoset [10] and human [11].

*Apodemus sylvaticus *is a common rodent species found throughout Europe. *A. sylvaticus *has a gross appearance similar to that of the laboratory mouse. The rearing conditions are also quite similar to those of the mouse. However, the superficial resemblance between *A. sylvaticus *and the laboratory mouse belies the rather deep evolutionary divide separating these two species. The combination of these properties--that is, the similar rearing conditions and large evolutionary divergence--makes *A. sylvaticus *highly attractive as a potential model organism that could perhaps complement the mouse in many studies. Unlike the mouse, however, there is a dearth of knowledge and reagents related to *A. sylvaticus*. One major step in filling this gap is the generation of ES cells for this species. Recently, we reported the successful establishment of an ES cell line from *A. sylvaticus *[12], named AS-ES1 cells. This cell line has proliferated continuously for over 6 months with a normal karyotype. It expresses a variety of markers associated with the undifferentiated state and has the ability to produce lineages of all three germ layers in vitro and in vivo. However, there are some characteristic differences between AS-ES1 and mouse ES cells. For example, AS-ES1 cells do not express stage specific embryonic antigen-1 (SSEA-1), whereas mouse ES cells do. Furthermore, the AS-ES1 cell line proliferates faster than specific mouse ES cell lines. Therefore, as a new species of ES cell line, the basic characteristics of AS-ES1 cells need to be studied further, including specific lineage differentiation.

Mouse ES cells were first established in 1981. Since then, many studies have been carried out regarding the three lineages differentiation of mouse ES cells in vitro. For mesodermal differentiation of mouse ES cells in vitro, different research groups have generated a variety of cell types, such as adipocytes [13,14], osteoblasts [15-19], chondrocytes [20-22] and cardiomyocytes [23,24], among others. Through this research, some pivotal agents that play an important role in the process of mesodermal differentiation of mouse ES cells have been discovered. However, it was not known whether those agents and differentiation methods could work with AS-ES1 cells.

Herein we report that AS-ES1 cells treated with retinoic acid (RA) or 5-azacytidine (5-AZA) at the embryoid body (EB) stage, with the addition of various specific factors and reagents to the medium during EB attachment, generated multiple mesodermal lineages in vitro, including adipocytes, osteoblasts, chondrocytes and cardiomyocytes.

## Results

### AS-ES1 cells maintained their undifferentiated state and generated embryoid bodies (EBs) in vitro

**Figure 1:**
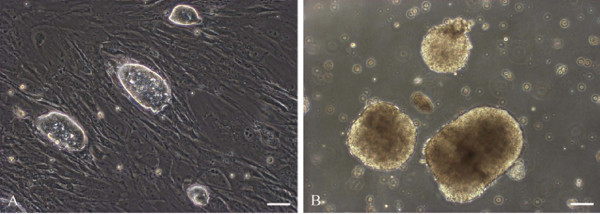
***Apodemus sylvaticus *ES cell and embryoid body**. Phase-contrast images of *Apodemus sylvaticus *ES cell colonies (**A**) and embryoid bodies after 7 days of formation (**B**). Scale bar, 100 μm.

AS-ES1 cells maintained their undifferentiated state when cultured on mouse embryonic fibroblast (MEF) cells. The ES clones had a dome-like shape with smooth and clearly defined borders (Fig. 1A). The ES cells between P50 and P60 were dissociated to single cells for forming EBs. After hanging drop culture for 2 days, 80% of the ES cell clumps began to organize into three-dimensional aggregates. After 7 days of culture, the aggregates grew into spherical EB-like structures with a uniform size and central transparency (Fig. 1B).

### AS-ES1 cells were capable of in vitro adipogenesis, osteogenesis and chondrogenesis

**Figure 2:**
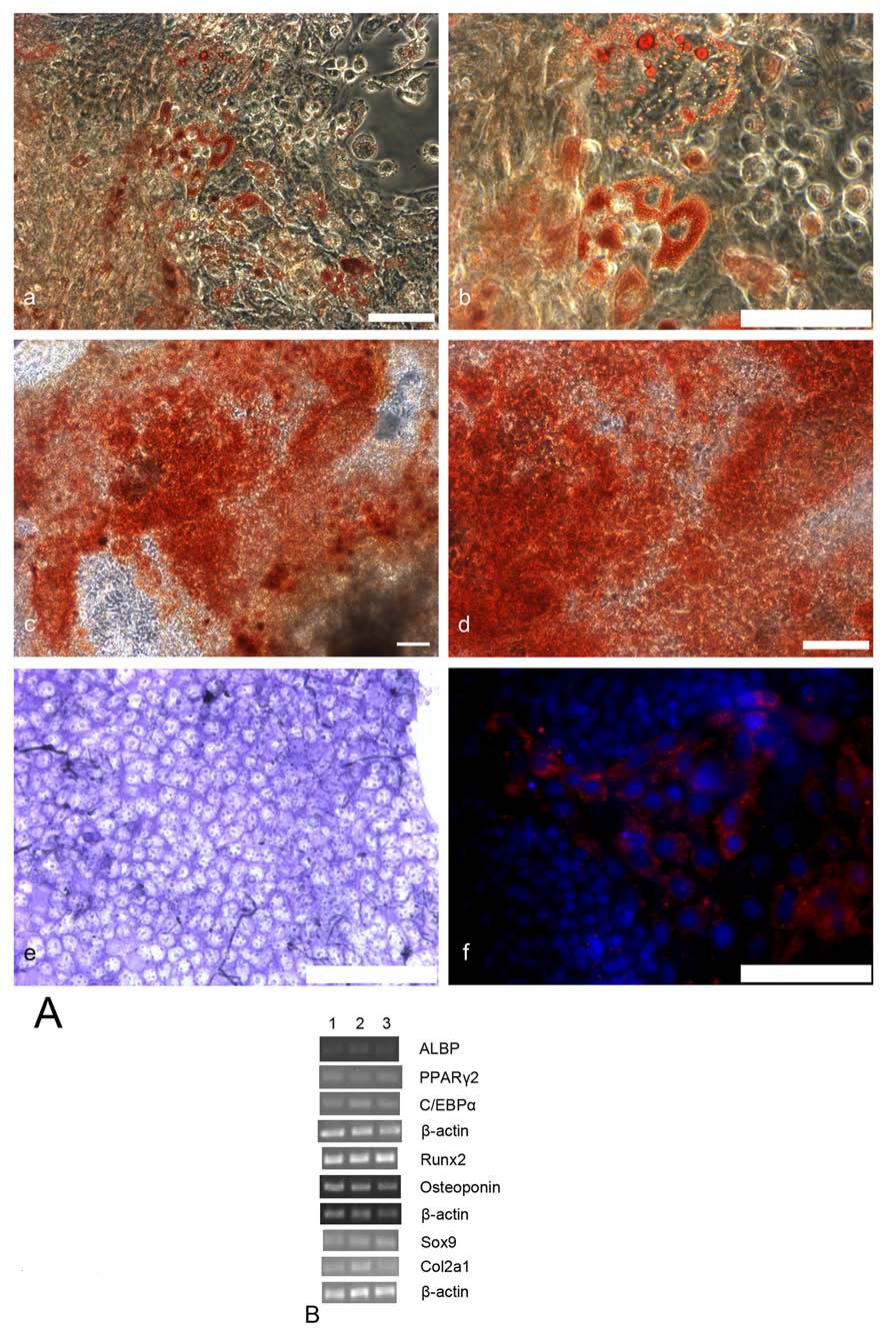
**Adipogenesis, osteogenesis and chondrogenesis of *Apodemus *ES cells in vitro**. Histochemistry and immunofluorescence staining illustrates adipogenesis, osteogenesis and chondrogenesis of *Apodemus *ES cells. (**A**) EB outgrowths stained by Oil Red O, (**a**, **b**) Alizarin Red, (**c**, **d**) or Toluidine Blue (**e**), EB outgrowths simultaneously stained for positive collagen II expression (**f**, **Red: anti-collagen II antibody; Blue: Hoechst 33342**). (**B**) Representative image of RT-PCR of adipogenesis, osteogenesis and chondrogenesis specific genes, expressed on day 28 of differentiation. **Numbers 1, 2 and 3 **represent three independent experiments in adipogenesis, osteogenesis and chondrogenesis. Scale bar, 100 μm.

Adipocytes, osteoblasts and chondrocytes derived from AS-ES1 cells emerged in EB outgrowths following in vitro differentiation. Adipocytes contain lipid droplets that can be easily visualized with oil red O staining. EB outgrowth cells stained with oil red O were observed as a large adipocyte colony that developed in the outgrowth of the aggregate (Fig. 2A, a and 2A, b). Calcium deposition, detected by alizarin red, also occurred in EB outgrowth areas (Fig. 2A, c and 2A, d). These calcium nodules are secreted by osteoblasts. Light- to dark-red/purple regions became noticeable within the outgrowth cells with toluidine blue staining. This is indicative of cartilage-like extracellular matrix accumulation. The cartilage nodules consisted of well separated round cells, and large extracellular spaces were stained metachromatically with toluidine blue (Fig. 2A, e). To further confirm chondrogenesis, EB outgrowths were stained with an anti-collagen IIantibody. The extracellular matrix positively expressed collagen II (Fig. 2A, f).

The percentage of positive cells stained by oil red O, alizarin red, toluidine blue or anti-collagen IIantibody differed. The proportion of positive cells in chondrogenesis was the greatest (toluidine blue, 20.69 ± 1.16%; anti-collagen IIantibody, 30.46 ± 1.87%), whereas the proportion of positive cells in adipogenesis (8.98 ± 0.89%) was similar to that in osteogenesis (9.05 ± 0.88%).

Expression of specific genes for adipogenesis, osteogenesis and chondrogenesis was investigated by Reverse Transcription-Polymerase Chain Reaction (RT-PCR) (Fig. 2B). We analyzed expression levels for genes including the adipocyte lipid binding protein (ALBP), peroxisome proliferative-activated receptor γ2 (PPARγ2), CCAAT/enhancer binding protein α (C/EBPα), Runx2, Osteopontin, Sox9 and Col2a1. RT-PCR of β-actin and undifferentiated AS-ES1 cells were included as positive and negative controls, respectively.

### Generation of cardiomyocytes by AS-ES1 cells

**Figure 3:**
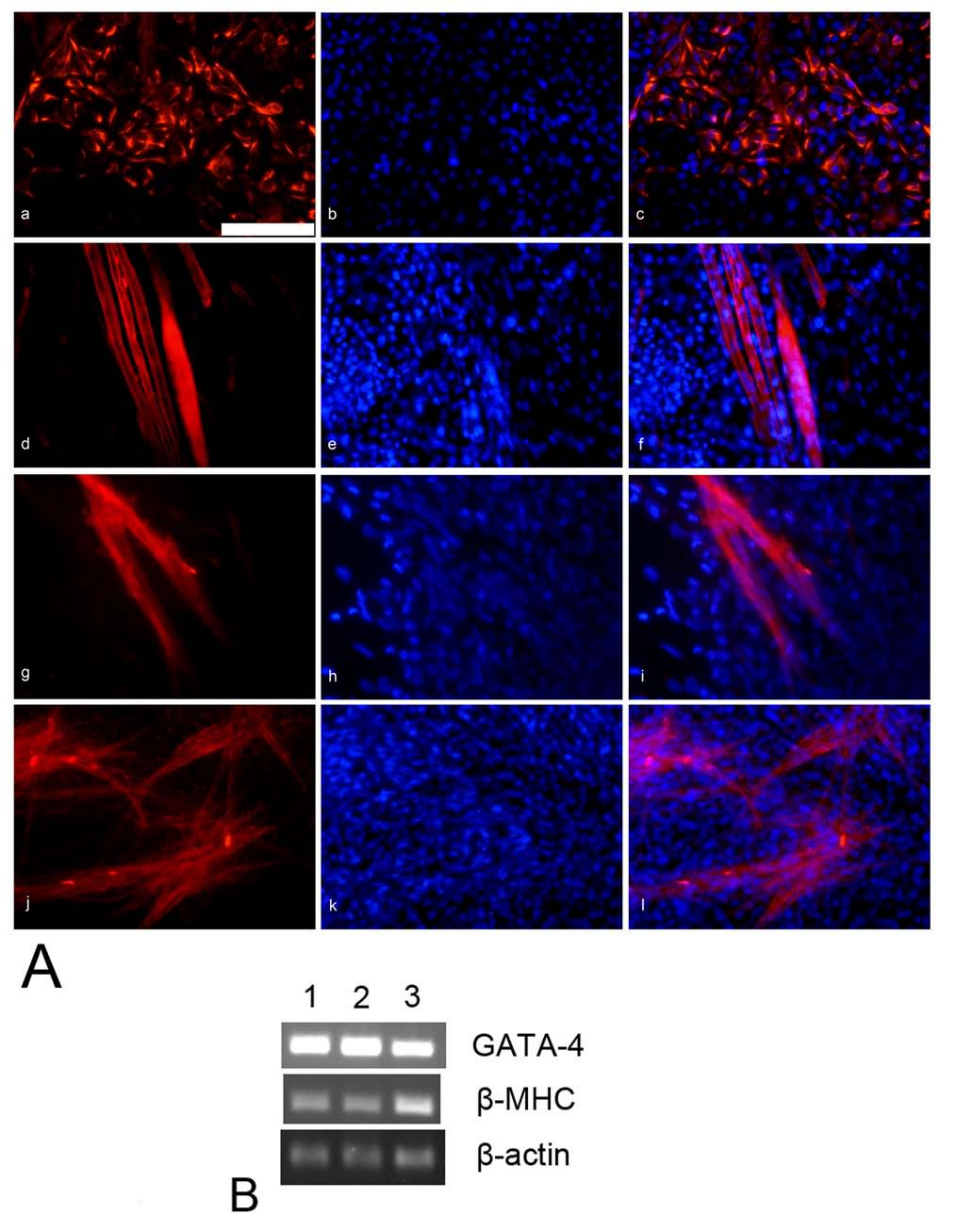
**Cardiomyocytes derived from *Apodemus *ES cell in vitro**. Immunofluorescence staining illustrated cardiomyocytes derived from *Apodemus *ES cells. (**A**) Cells were stained with anti-desmin (**a**), anti-sarcomeric α-actinin (**d**), anti-cardiac myosin heavy chain (**g**) or anti-cardiac troponin I (**j**) antibodies as indicated. Hoechst 33342 was used to stain nuclei (**b, e, h **and **k**). **Figures c, f, i **and **l **are merged images. (**B**) Expression of cardiac markers (GATA-4 and β-MHC) was determined by RT-PCR. **Numbers 1, 2 and 3 **represent three independent experiments in cardiomyocyte formation. Scale bar, 100 μm.

Besides adipocyte, osteoblast and chondrocyte generation, under cardiomyocyte differentiation conditions beating cells were observed in EB outgrowths as early as 16 days after plating. As differentiation proceeded, more and more beating cells could be observed by microscopy (see Additional file 1: Movie for the beating cells). Twenty-one days after plating, we performed immunostaining for cardiac muscle markers. AS-ES1 cell-derived cardiomyocytes were positively stained with anti-desmin (Fig. 3A, a), anti-sarcomeric α-actinin (Fig. 3A, d), anti-cardiac myosin heavy chain (MHC) (Fig. 3A, g) and anti-cardiac troponin I (Fig. 3A, j) antibodies. The cardiomyocytes exhibited striations and staining patterns of cytoplasmic localization that are characteristic of the sarcomeric organization of muscle cells. The numbers of positive cells stained by the four antibodies were analyzed. The proportions were as follows: anti-desmin antibody, 25.19 ± 2.19%; anti-sarcomeric α-actinin antibody, 10.12 ± 1.18%; anti-MHC antibody, 15.16 ± 1.41% and anti-cardiac troponin I antibody, 16.97 ± 0.92%. Expression of other cardiac markers GATA-4 and β-myosin heavy chain (β-MHC) was also determined by RT-PCR (Fig. 3B).

## Discussion

In our previous research, AS-ES1 cells spontaneously differentiated into cells of three germ lineages in vitro and produced healthy chimeras bearing extensive contributions in all major organs [12]. As a new ES cell line, AS-ES1 cells require more experimentation to discover their features. In this paper, we focused on the formation of specific mesodermal cells, derived from AS-ES1 cells, cultured with various factors and reagents in vitro, including dexamethasone, β-glycerophosphate, ascorbic acid, insulin and transforming growth factor (TGF) β1.

First, we treated EBs of AS-ES1 cells with RA for 3 days, which was previously reported to induce the development of mesenchymal cell lineages from mouse ES cells [13,25]. The distinctive effect of RA on ES cell differentiation is uncertain. It was reported that RA enhances neural crest generation, a major source of mesenchymal elements in RA-treated EBs, but not mesoderm development directly, to drive the formation of mesodermal type cells [18]. However, RA suppresses cardiomyocyte formation [13,25]. Thus, 5-AZA, which has previously been employed for the formation of cardiomyocytes derived from ES cells [24,26], was used to replace RA for cardiomyocyte differentiation in this study.

After EB formation, during the EB attachment stage, various factors and reagents, such as dexamethasone, β-glycerophosphate and TGFβ1, have been used to enhance differentiation. These factors and reagents were important elements for mesoderm differentiation of mouse ES cells into adipocytes, osteoblasts or other types of mesodermal cells [13,15-18]. In our experiment, we obtained four types of mesodermal cells separately by applying similar factors and reagents. With cardiomyocyte differentiation, we preferred a simple compound, ascorbic acid, rather than 5-AZA, because of 5-AZA cytotoxicity. The Takahashi group [23] screened a broad range of compounds and determined that ascorbic acid markedly increased the efficiency of cardiac differentiation from mouse ES cells. In addition, Sato's group [27] demonstrated that ascorbic acid enhanced the differentiation of ES cells into cardiomyocytes through collagen synthesis. From quantitative data, we could estimate that the yield of the four cell types--adipocytes, osteoblasts, chondrocytes and cardiomyocytes--was 10-30% of the total cell number. The proportions were similar to those of the four cell types derived from mouse ES cells [18].

## Conclusions

This study demonstrated that AS-ES1 cells could be induced to differentiate into mesodermal lineages by a combination of growth factors and chemicals and provides more information to further understand the differentiation characteristics of AS-ES1 cells.

## Methods

### AS-ES1 Cell Culture

AS-ES1 cells were cultured on MEF cells mitotically inactivated by 55-gray γ-irradiation, as previously described [12]. The ES cell medium contained high-glucose DMEM (Hyclone Laboratories Inc., Logan, UT, USA) supplemented with 10% fetal bovine serum (Hyclone), 10^3 ^units/ml recombinant murine leukemia inhibitory factor (LIF) (Chemicon International Inc., Temecula CA, USA), 0.1 mM b-mercaptoethanol (Sigma, St. Louis MO, USA), 1 mM sodium pyruvate (Gibco/Invitrogen, Grand Island NY, USA), 1× nonessential amino acids (Sigma) and 1× penicillin/streptomycin (Gibco/Invitrogen). The AS-ES1 cells were kept at 37°C in a humidified atmosphere with 5% CO_2 _and were passaged every 2-3 days.

### Embryoid Body (EB) Formation and Differentiation Media

AS-ES1 cells were dissociated with 0.25% trypsin/1 mM EDTA (Gibco/Invitrogen) and resuspended in EB medium. This medium consisted of high-glucose DMEM, 20% fetal bovine serum, 1× nonessential amino acids and 1× penicillin/streptomycin. Hanging drops containing 4-5 × 10^3 ^cells in 20 μl of EB medium were maintained for 2 days on the lid of bacteriological dishes filled with phosphate-buffered saline (PBS). The EBs were then transferred into bacteriological dishes and maintained for 3 days in suspension in EB medium supplemented with either 10^-7 ^M all-trans retinoic acid (RA, Sigma) [13,18] for adipo-, osteo- and chondrogenesis, or 10^-6 ^M 5-azacytidine (5-AZA, Sigma) [24] for cardiomyocyte generation. The medium was changed daily. EBs were maintained for 2 more days in suspension in EB medium and were then allowed to settle onto 2% gelatin-coated plates in the presence of various differentiation media. The differentiation media contained high-glucose DMEM, 10% fetal bovine serum and 1× penicillin/streptomycin supplemented with the following reagents: 1 μm/L dexamethasone, 0.2 mM indomethacin, 0.5 mM 3-isobutyl-1-methylxanthine (IBMX) and 10 μg/ml insulin for adipogenesis [14,28]; 0.1 μm/L dexamethasone, 0.2 mM ascorbic acid and 10 mM β-glycerophosphate for osteogenesis [15,17]; 10 ng/ml transforming growth factor (TGF) β1 and fetal bovine serum reduced to 3% for chondrogenesis [18,29]; and 0.4 mM ascorbic acid for cardiomyocyte formation [23]. TGFβ1 was purchased from PeproTech EC (London, UK) and the other reagents were obtained from Sigma. All of the differentiation media were changed every three days.

### Histochemical Staining

The day of EB initial formation represented day 0. EB outgrowths induced for 28 days were fixed by 10% neutral formaldehyde for 20 min, washed with dH_2_O and then processed as follows for the different stains. The results of the histochemical staining were investigated under an inverted microscope (Olympus, Japan).

Oil Red O staining: samples were incubated with 2% oil red O (Sigma) in 60% iso-propyl alcohol for 40 min. To avoid non-specific staining, the samples were incubated with 60% iso-propyl alcohol for 2 min and washed with dH_2_O four times.

Alizarin Red staining: specimens were incubated with 40 mM alizarin red (Sigma) in dH_2_O for 20 min and washed with dH_2_O four times.

Toluidine Blue staining: specimens were incubated with 1% toluidine blue (Sigma) for 2 h, washed with 95% ethanol one time and rinsed in dH_2_O four times.

### Immunofluorescence Staining for Cells

EB outgrowth cells were fixed with 4% paraformaldehyde in PBS, permeabilized by 0.1% Triton X-100 for 30 min and blocked with 10% normal goat serum in PBS for 1 h. The cells were first incubated with primary antibodies against desmin (Neomarkers, Fremont CA, USA, 200× dilution), sarcomeric α-actinin (Sigma, 400× dilution), cardiac myosin heavy chain (Chemicon, 100× dilution), cardiac troponin I (Chemicon, 100× dilution) or collagen II (Rockland Inc., Gilbertsville PA, USA, 200× dilution) at 4°C overnight, followed by incubation with Cy3-conjugated goat anti-mouse IgG or goat anti-rabbit IgG (Southern Biotech, Birmingham AL, USA) for 1 h at room temperature. The specimens were mounted in a vectashield with Hoechst 33342 (Sigma) and observed under a fluorescence microscope (Olympus).

### Reverse Transcription-Polymerase Chain Reaction (RT-PCR) Assay

Total RNA from EB outgrowths was extracted using TRIzol (Gibco/Invitrogen). The first strand complementary DNA was synthesized using Sensiscript Reverse Transcriptase (Qiagen GmbH, Hilden, Germany) followed by PCR amplification. The primer sequences and PCR product sizes were: β-actin (F-AGAAGATCTGGCACCACACC, R-TACGACCAGAGGCATACAGG; 198 bp), ALBP (F-TTGGTCACCATCCGGTCAGA, R-TTCCACCACCAGCTTGTCAC; 207 bp), PPARγ2 (F-ATCATCTACACGATGCTGGCC, R-CTCCCTGGTCATGAATCCTTG; 80 bp), C/EBPα (F-CGCAAGAGCCGAGATAAAGC, R-GCGGTCATTGTCACTGGTCA; 80 bp), Runx2 (F-CCTGAACTCTGCACCAAGTC, R-GAGGTCGCAGTGTCATCATC; 234 bp), Osteopontin (F-TCTCCTTGCGCCACAGAATG, R-TCCTTAGACTCACCGCTCTT; 398 bp), Sox9 (F-GCAGACCAGTACCCGCATCT, R-CTCGCTCTCGTTCAGCAGC; 80 bp), Col2a1 (F-CCGTCATCGAGTACCGATCA, R-CAGGTCAGGTCAGCCATTCA; 228 bp), GATA-4 (F-CTGTCATCTCACTCTGGGCA, R-CCAAGTCCGAGCAGGAATTT; 256 bp), β-MHC (F-ACCCCTACGATTATGCG, R-GTGACGTACTCGTTGCC; 319 bp). The amplification fragments were analyzed on a 2% agarose gel. Imaging and scanning densitometry quantification were performed on a Kodak image station 2000R (Kodak, Rochester NY, USA).

### Positive cell staining counts and statistical analyses

All experiments of adipogenesis, osteogenesis, chondrogenesis and cardiomyocyte formation were performed at least three times. Data for percentage of positive cells were expressed as mean ± standard deviation and analyzed by Student's t test. A value of P < 0.05 was considered statistically significant.

## Authors' contributions

TW performed the experiments, analyzed the data analysis and drafted the manuscript; FFM performed experiments and data analysis; WYL and WQL performed cell culture experiments; WHY and ZFW performed immunostaining; LRZ carried out RT-PCR; JLZ and JN participated in provision of study material, collection and/or assembly of data. XMZ performed the statistical analysis. BTL participated in study design. APX designed the experiments and drafted the manuscript. All authors read and approved the final manuscript.

## Supplementary Material

Additional file 1 **Movie for the beating cells**. At 21 days after plating, under cardiomyocyte differentiation conditions, beating cells in EB outgrowths were photographed by Olympus phase-contrast microscope. Click here for file
